# The Neural Code for Auditory Space Depends on Sound Frequency and Head Size in an Optimal Manner

**DOI:** 10.1371/journal.pone.0108154

**Published:** 2014-11-05

**Authors:** Nicol S. Harper, Brian H. Scott, Malcolm N. Semple, David McAlpine

**Affiliations:** 1 UCL Ear Institute, University College London, London, United Kingdom; 2 Redwood Center for Theoretical Neuroscience, University of California, Berkeley, CA, United States of America; 3 Laboratory of Neuropsychology, National Institute of Mental Health, National Institute of Health, Bethesda, MD, United States of America; 4 Center for Neural Science, New York University, New York, NY, United States of America; University of Southern California, United States of America

## Abstract

A major cue to the location of a sound source is the interaural time difference (ITD)–the difference in sound arrival time at the two ears. The neural representation of this auditory cue is unresolved. The classic model of ITD coding, dominant for a half-century, posits that the distribution of best ITDs (the ITD evoking a neuron’s maximal response) is unimodal and largely within the range of ITDs permitted by head-size. This is often interpreted as a place code for source location. An alternative model, based on neurophysiology in small mammals, posits a bimodal distribution of best ITDs with exquisite sensitivity to ITDs generated by means of relative firing rates between the distributions. Recently, an optimal-coding model was proposed, unifying the disparate features of these two models under the framework of efficient coding by neural populations. The optimal-coding model predicts that distributions of best ITDs depend on head size and sound frequency: for high frequencies and large heads it resembles the classic model, for low frequencies and small head sizes it resembles the bimodal model. The optimal-coding model makes key, yet unobserved, predictions: for many species, including humans, both forms of neural representation are employed, depending on sound frequency. Furthermore, novel representations are predicted for intermediate frequencies. Here, we examine these predictions in neurophysiological data from five mammalian species: macaque, guinea pig, cat, gerbil and kangaroo rat. We present the first evidence supporting these untested predictions, and demonstrate that different representations appear to be employed at different sound frequencies in the same species.

## Introduction

**Figure 1:**
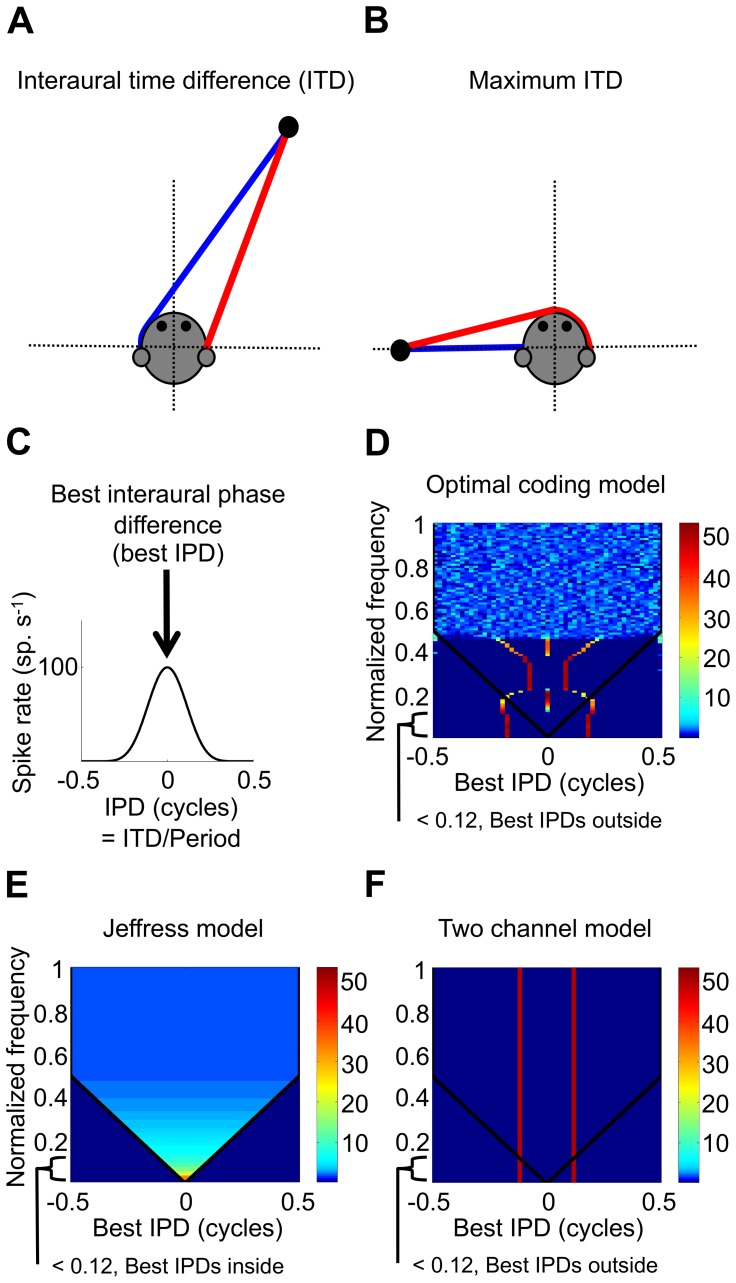
Models predicting the distribution of best ITDs. (A) Illustration of an ITD from a sound source (B) Illustration a sound source near the interaural axis (horizontal dotted line) having maximum ITD. In A–B red and blue lines are the shortest paths from the sound source (black dot) to the ears. (C) A illustration of a model rate-vs-IPD function and best IPD. IPD is ITD as a proportion of the period of the sound frequency. (D) Optimal-coding model: complex frequency dependent distribution. (E) Jeffress model: homogeneous distribution or unimodal distribution of best IPDs at all frequencies, largely within the physiological range. (F) Two-channel model, bimodal at all frequencies. In Figure (D–F), solid black line, maximum IPD, white line, limit of IPD sensitivity, color, percentage of neurons at a given frequency with that best IPD. The ordinate is the sound frequency normalized with respect to the reciprocal of the maximum ITD (i.e. sound frequency as a proportion of 1/maximum ITD).

For many species, including humans, a major cue for sound-source lateralization is the interaural time difference (ITD), the difference in arrival time of a sound at the two ears [1], [2]. Sound from a source takes longer to reach the far ear than the near ear, resulting in an ITD, with the exact ITD depending on the position of the sound source (Figure 1A). The classic model of the neural representation of ITD, developed by Jeffress [3], proposes an array of coincidence-detector neurons fed by a series of internal delay lines originating from each ear. Each neuron generates a maximal response at its ‘best ITD’, when the difference in internal delay compensates the external ITD, bringing the neuron’s inputs into coincidence. The Jeffress model posits that best ITDs are distributed within the “physiological range” of ITDs, generated by the size and shape of the head. This range is bounded by the maximum ITD, found for sound sources near the interaural axis (Figures 1B). Most instantiations of the Jeffress model posit a unimodal or uniform distribution of best ITDs centred at zero ITD [4]–[10], designed to account for the better spatial acuity observed for frontal locations. This ‘centrality' in the neural representation of ITD was first suggested by Sayers and Cherry [11], and also proposed by Jeffress [12]. The Jeffress model is often envisaged as an explicit place code for ITD, and commonly assumed to apply at all sound frequencies.

Basic features of the Jeffress model are generally considered consistent with anatomical and physiological features of the barn-owl auditory brain [13]–[15] and were also considered to hold for mammals [16]. More recently, however, neural recordings from several mammalian species report a bimodal distribution of best ITDs, with the best ITDs of many neurons, counter-intuitively, lying beyond the physiological range. This suggested a model in which ITDs are encoded by means of the relative firing rates of opposing neural populations [17]–[25]. The disparate features of the Jeffress and two-channel models were recently unified under an optimal-coding model derived from the principle of efficient coding [26]. The optimal-coding model predicts that the uniform representation suggested by Jeffress provides for optimal ITD discrimination in species with relatively large heads (and therefore large maximum ITDs) and at relatively high sound-frequencies, whereas the bimodal representation of the two-channel model confers an advantage in species with small head-sizes, and for larger species at low sound-frequencies. In addition to offering a unifying explanation for the range of experimental observations, the optimal-coding model predicts that for many mammalian species, including humans, both forms of representation will be evident, depending on the sound frequency. It also predicts additional, novel representations that conform to neither the Jeffress nor two-channel models. Here, we assess experimental data from five mammalian species, and demonstrate evidence supporting these predictions. In particular, we demonstrate that, for many species, ITD is encoded by different neural representations at different sound frequencies.

## Results

Experimentally recorded distributions of best ITDs were examined as a function of sound frequency for five mammalian species with different head sizes, and compared to the distributions predicted by the optimal-coding model, the Jeffress model, and the two-channel model. Figure 1D illustrates the general predictions of the optimal-coding model. In this and subsequent figures, ITD is plotted as interaural phase difference (IPD) - the ITD as a proportion of the period of the sound frequency (provided on the ordinate). In Figures 1D–F, sound frequency is plotted normalized by 1/maximum ITD, and thus predictions for different species with different maximum ITDs constitute scaled versions of these Figures (with slight distortions if the relatively small fluctuations of maximum ITD with sound frequency are accounted for). The actual distributions would be expected to be substantially more diffuse than the predictions of the models, with distinct sub-populations of the models being peaks in the distribution of best IPDs in the data.

Broadly stated, the main untested prediction of the optimal-coding model is that, for intermediate and larger head-sizes, different neural representations will be observed at different sound frequencies in the same species. However, for species with smaller head-sizes, the optimal-coding model predicts only the two-channel representation to be present at all frequencies (as reported experimentally). To this end, for rigorous cross-species comparisons, data sets for these smaller species are also subject to the same analysis as for the other, larger, species. Experimental data we recorded from guinea-pigs and macaques are analysed. In addition, new analyses of data sets extracted from previously published studies of ITD sensitivity in the kangaroo rat, gerbil, and cat are performed. For the cat, guinea pig, and gerbil data, the frequencies at which the best IPDs were measured were the best frequency (or a similar frequency tuning measure) of each neuron (best frequency, BF, is the frequency that elicits the greatest firing rate for a given sound intensity). For the macaque and kangaroo rat data, best IPDs were measured for a small number of stimulating sound frequencies within a neuron’s frequency tuning range. For these data sets, either case is expected to be reasonable for testing the broad predictions of the optimal-coding model (see Methods). For each species, the data are represented in the form of a 2D histogram, plotting the number of neurons with particular best IPDs as a function of frequency.

For data for each of the five species, the form of the distributions of best IPDs over sound frequency was analyzed, sufficient data permitting. The results were then compared to the predictions of the optimal-coding model, as well as the Jeffress model and the two-channel model. Figures 1E–F illustrate the predicted distributions of best IPD as a function of best frequency for the Jeffress model (for a uniform distribution) and the two-channel model, respectively, if predictions from these models were to hold over the entire frequency range.

Specifically, the following questions were addressed in the analysis (see Methods for details):

Q1) At low frequencies (normalized frequency below ∼0.12), does the distribution of best IPDs fall largely outside the physiological range (i.e. is it two-channel), as predicted by the optimal-coding model? Although this prediction has been tested in some small mammals, it has not been tested in a systematic manner across species.

Q2) At intermediate frequencies (normalized frequency ∼0.12 to ∼0.5), do the data indicate the novel distributions of best IPDs predicted by the optimal-coding model, with a central peak in the distribution in the lower intermediate frequencies and above that a bimodal distribution within the physiological range? Neither the Jeffress model nor two-channel models predict this. This prediction is as yet untested.

Q3) At high frequencies (normalized frequency above ∼0.5), do the data tend toward a uniform or unimodal best-IPD distribution (i.e. is the distribution Jeffress-like), as predicted by the optimal-coding model? This prediction has not yet been examined in mammals.

### The distribution of best ITDs is bimodal in small mammals, but shows frequency-dependent ITD representation in intermediate-sized mammals

#### Small mammals

**Figure 2:**
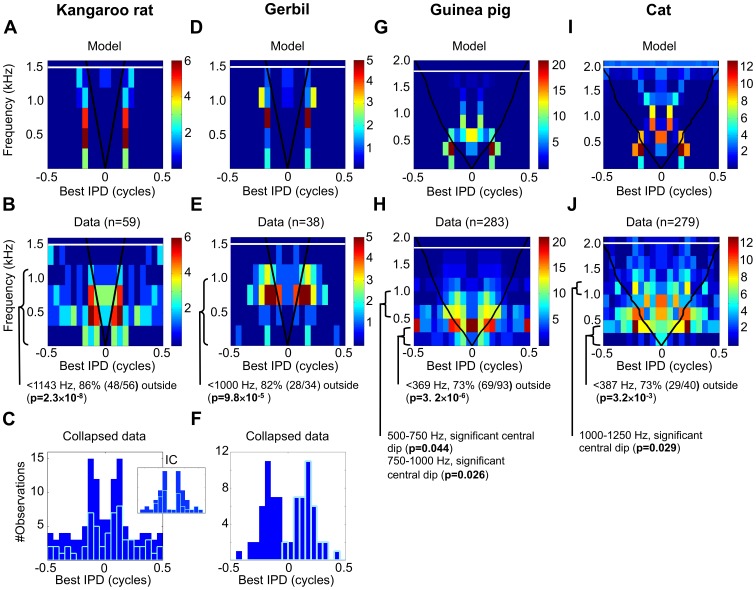
Best delay distributions for small and intermediate-sized mammals. (A) Predicted distributions of best IPDs by the optimal-coding model for the kangaroo rat (max. ITD, ∼105 µs, highest frequency with ITD sensitivity ∼1500 Hz). IPD is ITD is a proportion of the period of the sound frequency on the ordinate. Black line, maximum IPD that limits the physiological range, white line, limit of IPD-sensitivity, color, number of best IPDs in an IPD bin for a given frequency band. The predictions uses the same size frequency and IPD bins as the corresponding data in Figure 2B, and each frequency band has been scaled to have the same maximum as the corresponding frequency band in the data. (B) Best ITD data from the Crow et al. [18] kangaroo rat SOC study (59 data points successfully extracted), converted from ITD to IPD, mirrored, and then re-plotted as a 2D histogram using 300-Hz frequency bins and 0.05 cycle IPD bins. Format a Figure 2A. (C) Best IPD data from Figure 2B collapsed over frequency, solid blue bars are mirrored data, empty light blue bars unmirrored data. Inset figure plots data from the kangaroo rat inferior colliculus (IC) in the midbrain [25], showing number of neurons with a given characteristic delay (similar to best ITD). The abscissa on the inset figure spans −7.5 ms to 7.5 ms, with an ordinate spanning 0 to 14 neurons. (D) Predicted distributions of best IPDs by the optimal-coding mode for the Mongolian gerbil (max. ITD, ∼120 µs, highest ITD-sensitive frequency ∼1500 Hz). Format as Figure 2A. (E) Best ITD data from Pecka et al. [28] Mongolian gerbil medial superior olive study (38/40 data points successfully extracted), converted from ITD to IPD, mirrored, and re-plotted as a 2D histogram using 300 Hz frequency bins and 0.05 cycle IPD bins. Format as Figure 2A. (F) Data in Figure 2E collapsed over frequency. Format as Figure 2C. (G) Predicted distributions of best IPDs by the optimal-coding model for the guinea pig (max. ITD, 245–330 µs, highest frequency with ITD sensitivity ∼1800 Hz). Format as Figure 2A. (H) 260 best IPDs from the guinea pig inferior colliculus mirrored and re-plotted as a 2D histogram using 250 Hz frequency bins and 0.05 cycle IPD bins. Format as Figure 2A. (I) Predicted distributions of best IPDs by the optimal-coding model for the cat (max. ITD, 250–325 µs, highest frequency with ITD sensitivity ∼2000 Hz). Format as Figure 2A. (J) Combined best IPD data from the Hancock and Delgutte [19] and the Joris et al. [37] cat inferior colliculus studies (86/107 and 193/219 data points successfully extracted respectively) converted from ITD to IPD, mirrored, and re-plotted as a 2D histogram using 250 Hz frequency bins and 0.05 cycle IPD bins. Format as Figure 2A.

Two small mammals for which the distribution of best ITDs has been reported are the banner-tailed kangaroo rat (*Dipodomys spectabilis*) and the Mongolian gerbil (*Meriones unguiculatus*). These species have maximum ITDs of 105 µs [25] and 120 µs [27] respectively. The predictions of the optimal-coding model for the kangaroo rat and gerbil (Figure 2A and 2D, respectively) are that the majority of best IPDs lie beyond the physiological range, and that best IPDs are distributed bimodally at all but the highest frequencies. Previous studies have argued for the existence of a bimodal distribution of ITDs in these species, consistent with the two-channel model and the predictions of the optimal-coding model. In the kangaroo rat, Crow et al. [18] measured best ITDs as a function of frequency for neurons recorded from the superior olivary complex of the hindbrain, the presumed site of primary binaural integration. In the gerbil, Pecka et al. [28] measured best ITD as a function of best frequency in the medial superior olive, the dominant ITD-sensitive nucleus of the superior olivary complex. Here, we re-analyze this data in order to compare them in a consistent manner with data obtained from other species.

The data from the kangaroo rat of Crow et al. [18] are re-plotted in Figure 2B, with the distribution of best IPDs plotted for a number of frequency bands as a ‘mirrored’ 2D-histogram. Mirroring assumes symmetry in the neural representation, with each neuron having an opposing neuron with a best ITD of equal magnitude but opposite sign. Recall that IPD equates to ITD as a proportion of the period of the sound frequency along the ordinate. Figure 2C plots the same data as Figure 2B, but collapsed across sound frequency. Figures 2E re-plots the data obtained from histologically identified medial superior olive neurons by Pecka et al. [28], using the same form of mirrored 2D-histogram employed in Figure 2B. Figure 2F shows the same data as in 2E, again collapsed across sound frequency. Examining question Q1 - whether the distribution of best IPDs falls largely outside the physiological range for frequencies below the ‘specific’ frequency (normalized frequency 0.12, in kangaroo rat, 1143 Hz, in gerbil, 1000 Hz), the optimal-coding model predicts that a greater number of best IPDs does indeed lie beyond the physiological range (i.e. outside the black lines in Figures 2A–B and 2D–E) than within. The null hypothesis, consistent with the Jeffress model, postulates the opposite. For the kangaroo rat, 86% (48/56) of best IPDs below the specific frequency lie beyond the physiological range. This proportion is significant (p = 2.3×10^−8^, one-tailed binomial test), and thus the null hypothesis is rejected. Employing the same analysis for the gerbil, 82% (28/34) of the best IPDs below the specific frequency lie beyond the physiological range. Again, this is significant (p = 9.8×10^−5^, one-tailed binomial test), and once more the null hypothesis is rejected. With respect to bimodality in the distribution of best IPDs, Figures 2B–C and 2E–F clearly indicate two distinct sub-populations across the entire frequency range for which data exist. This analysis demonstrates that, in small mammals, the distributions of best IPDs are consistent with the optimal-coding model: a two-channel code with distributions of best IPDs lying outside the physiological range (Q1 confirmed). Similar data are also found for recordings made in the inferior colliculus of the midbrain of the kangaroo rat [25], which receives direct input from the medial superior olive. This midbrain data is shown in the inset to Figure 2C, indicating that the form of the distribution is not specific to the brain centre from which the recordings were made.

The presence of an emerging central population is not assessed for these small mammals because there are insufficient data at these frequencies (Q2). The optimal-coding model predicts that regions of uniform distribution of best IPDs do not exist for these small mammals and the data are consistent with this finding (confirming Q3).

#### Intermediate-sized mammals

In order to assess the untested predictions of the optimal-coding model, it is necessary to examine data obtained from animals with larger head sizes, as it is for these species that different representations of ITD are predicted to arise over different frequency ranges. Two intermediate-sized mammals are the guinea pig (*Cavia porcellus*) and the cat (*Felis catus*), with maximum ITDs around 300 µs [29], [30]. The maximum ITD varies slightly with sound frequency in both the cat (250–325 µs range [30]) and guinea pig (245–330 µs range [29]). We used these frequency-dependent maximum ITDs in our model predictions. For these medium-sized species the optimal-coding model (Figure 2G and 2I, guinea pig and cat, respectively) predicts that best IPDs show a distribution consistent with the two-channel model for the lowest sound frequencies, with peaks in the distribution lying beyond either side of the physiological range (Q1). Above the specific frequency (normalized frequency 0.12), however, the model predicts the existence of distinct sub-populations within the physiological range. At these intermediate frequencies, it predicts a central sub-population at zero IPD around 500 Hz, and then a bimodal distribution above that (Q2). For the highest sound frequencies for intermediate-sized mammals, the model predicts a uniform distribution. However, for both the guinea and the cat there are too few data at the highest frequencies to test for uniformity (Q3).

For the guinea pig, best IPDs as a function of best frequency were obtained from 283 neurons in the inferior colliculus of the midbrain. Although neural sensitivity to ITD appears first in the medial superior olive, ITD-sensitive neurons in midbrain structures such as the inferior colliculus, and forebrain areas such as auditory thalamus and cortex, tend to display similar ITD-tuning properties [24], [31], as we see in Figure 2C. Therefore, experimental data recorded from all these brain areas are useful in testing the various models. Figure 2H plots the guinea pig data as a mirrored 2D-histogram. For neurons with frequency tuning below the specific frequency of 369 Hz, the lowest frequency at which a sub-population of best IPDs within the physiological range appears in the optimal-coding model, 73% (69/94) of best IPDs lie outside the physiological range (p = 3.2×10^−6^, one-tailed binomial test). Thus, the null hypothesis is rejected and the presence of distributions consistent with the two-channel model, with most best IPDs beyond the physiological range is confirmed (Q1 confirmed).

Regarding Q2, the 250–500 Hz frequency band in Figure 2H demonstrates a previously un-noted central sub-population of best ITDs. Also, above approximately 500 Hz, two opposing subpopulations are apparent that lie within, rather than beyond, the physiological range. The frequency bands that included at least some of the intermediate frequency range were examined for the presence of a tight central dip at zero IPD in the distribution of best IPD (that is, whether there are fewer best IPDs of magnitude <0.075 cycles than the are of magnitude 0.075–0.15 cycles). This tight dip would be indicative of a bimodal representation within the physiological range, as predicted by the optimal-coding model (see Methods). No such dip at 250–500 Hz was evident (not significant (N.S), n = 50, one-tailed binomial test), consistent with the null hypothesis, and consistent with an interpretation that a central subpopulation of best IPDs exists within this band. However, significant dips in the 500–750 Hz and 750–1000 Hz bands (p = 0.044 and 0.026, n = 28 and 22, respectively, one-tailed binomial test) were evident, consistent with the existence of opposing subpopulations that lie within the physiological range in these bands. This distribution pattern is consistent with the predictions of the optimal-coding model, and not predicted by either the Jeffress model or the two-channel model (Q2 confirmed).

As guinea pigs appear to have few neurons sensitive to fine-structure ITDs above 1–1.3 kHz [21], [22], [32], the data are insufficient to assess Q2 for neurons tuned to frequencies above 1000 Hz, or the existence of uniformity in the distribution at the highest frequencies (Q3). In addition, one potential anomaly relates to the sub-population around 0.5 cycles in the 250–500 Hz band. In this data set, neurons with best IPDs near 0.5 cycles are almost all ‘trough-type’ neurons [33], showing a response *minimum* as their defining feature [34]–[36]. Since trough-type neurons (10–20% of ITD-sensitive neurons) were not included in the optimal-coding model, one would not expect their best IPDs to be predicted (see Methods).

The distribution of ITD detectors in the cat has previously been suggested to follow that suggested by the Jeffress model [16]. In Figure 2J, best IPDs as a function of best frequency, obtained from recordings made in the inferior colliculus of the cat [19], [37], are replotted as a mirrored 2D histogram. For neurons with frequency tuning below the specific frequency of 387 Hz, the majority of best IPDs, 73% (29/40) lie beyond the physiological range. Employing a one-tailed binomial test, this finding is significant (p = 3.2×10^−3^), and thus the null hypothesis is rejected (Q1 confirmed). In Figure 2J, in the range 500–1000 Hz, a clear central sub-population exists at 0 cycles IPD. In addition, qualitatively, it can be seen that this central subpopulation lies between opposing sub-populations that are most clearly present down to 250 Hz and up to 1250 Hz. Assessing evidence of a tight central dip in the distribution of best IPDs in the intermediate frequency range (as was performed for the data from the guinea pig), in the range 250–500 Hz, and where the central subpopulation is observed in the ranges 500–750 Hz and 750–100 Hz ranges, no evidence of a significant tight central dip is apparent (N.S. in all cases, n = 21, 18, and 23 respectively, one-tailed binomial test). However, a significant dip is observed in the range 1000–1250 Hz (p = 0.029, n = 14, one-tailed binomial test), consistent with the presence of opposing subpopulations lying within the physiological range in this frequency range (Q2 confirmed). This is qualitatively consistent with the predictions of the optimal-coding model - a central subpopulation above the specific frequency and, above that, two opposing subpopulations, with no central subpopulation. As with the guinea pig, too few data points exist above 1250 Hz to assess the form of the distribution over this frequency range (for Q2 and Q3). The central sub-population in the cat is at a higher frequency band than in the guinea pig, and we speculate as to why this might be in the Discussion. Overall, for both medium-sized species, the presence of different distributions at different frequencies is observed within a species, consistent with the previously untested predictions of the optimal-coding model.

### The distribution of best ITDs in a large primate also shows frequency-dependent ITD representation

**Figure 3:**
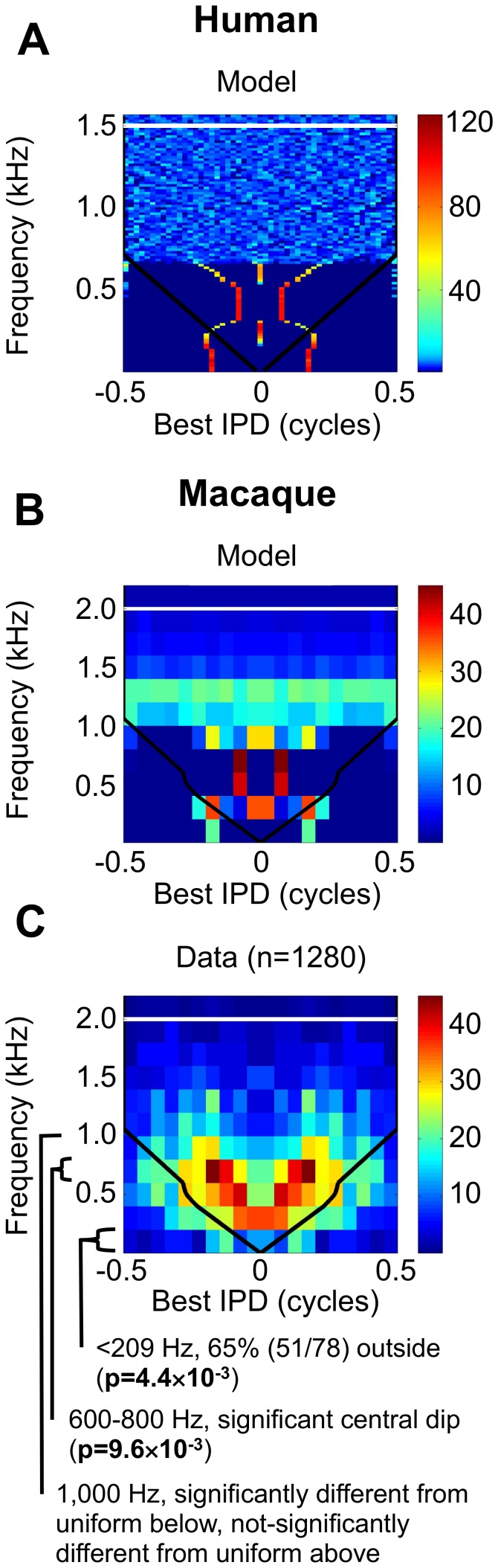
Best delay distributions for large primates. (A) The predicted distributions of best IPDs by the optimal-coding model for the human (max. ITD, ∼690 µs, highest frequency with ITD-sensitivity ∼1500 Hz). Color, number of best IPDs in an IPD bin for a given frequency band (200 best IPDs per band modelled), bin sizes as Figure 1D, otherwise format as Figure 2A. (B) The predicted distributions of best IPDs by the optimal-coding model for the macaque (max. ITD, 470–575 µs, max. ITD-sensitive frequency ∼2000 Hz). Format as Figure 2A. (C) Distribution of 1280 best IPDs, recorded from macaque auditory cortex, mirrored and then plotted as a 2D histogram using 200-Hz frequency bins and 0.05 cycle IPD bins. Format as Figure 2A.

The rhesus macaque monkey (*Macaca mulatta*) is a large primate. Its maximum ITD of approximately 500 µs [38], is the closest of all species in this study to the maximum ITD of 690 µs in humans [39]. The behavioural threshold for ITD discrimination in this species [40] is best over the range 500–1750 Hz, degrading above 2000 Hz. The similarity in macaques and humans in head size and the frequency range over which ITD sensitivity is observed, not to mention the close genetic relationship, make the macaque an excellent model from which to infer the representation of ITD in humans. Figure 3B plots the predicted distribution of best IPDs suggested by the optimal-coding model for the macaque, using a frequency-dependent maximum ITD (470–575 µs range, [38]). The general prediction for the optimal-coding model is that the representation of ITD should depend on the sound frequency: below the specific frequency (∼209 Hz), the majority of best IPDs lie beyond the physiological range, at low intermediate frequencies, a central subpopulation is evident, above which a bimodal distribution is again apparent (with best IPDs lying within the physiological range) and, at the highest frequencies (above ∼1000 Hz) a uniform distribution exists. The predictions for the macaque are similar to those of the human shown in Figure 3A.

The distribution of best IPDs was measured as a function of stimulating frequency, for neurons recorded from core auditory cortex in the macaque. The 1280 values of best IPD (see Methods) are plotted in the form of a mirrored 2D-histogram (Figure 3C - recall that IPD reflects ITD as a proportion of the period of the frequency). We first examined whether the majority of best IPDs below the specific frequency of 209 Hz in the macaque lie within or beyond the physiological range, establishing the null hypothesis that they lie within. From the data, 65% (51/78) of best IPDs reside beyond the physiological range (p = 4.4×10^−3^, one-tailed binomial test), and thus the null hypothesis is rejected (Q1 confirmed). Examining the form of the data, for the lowest sound frequencies (Figure 3C, 0–200 Hz), bimodality in the distribution of best IPDs is observed. At slightly higher frequencies (above 200 Hz), a single dominant central population emerges. At even higher frequencies (above 400 Hz), the data once more show opponent sub-populations of best IPDs, but here lying within the physiological range. Testing for a tight dip in best IPDs around zero IPD in each frequency band covering the intermediate frequency range (Q2), no evidence exists of a significant dip in the 200–400 Hz band, where the central subpopulation exists, or in the 400–600 Hz band (N.S. in both cases, n = 97 and 100 respectively, one-tailed binomial test). However, a significant central dip is evident in the 600–800 Hz band (p = 9.6×10^−3^, n = 89, one-tailed binomial test). There is no significant tight dip in the 800–1000 Hz and 1000–1200 Hz bands (N.S. in both cases, n = 45 and 25 respectively, one-tailed binomial test). This pattern of distinct sub-populations is similar to that predicted by the optimal-coding model which predicts that, with increasing sound frequency, the distribution of best IPDs shifts from one in which two opponent sub-populations lie beyond the physiological range, to one comprising a dominant central population, before reverting to one comprising two opponent sub-populations within the physiological range, before again changing pattern. Thus, once more, the data are qualitatively consistent with predictions of the optimal-coding model (Q2 confirmed).

With the large amount of macaque data available, it was also possible to test statistically the prediction of a uniform distribution of best IPDs over the whole cycle above a particular frequency (∼1000 Hz), but a non-uniform distribution over the range below it (Q3). To avoid sample size effects influencing the outcome (due to there being fewer neural recordings made at the higher frequencies), each of the 10 frequency bins was set so as to contain an equal number of samples (IPD values) per bin (any borderline data-points were randomly assigned to a side of the bin border). Kuiper’s Test was applied to each frequency band, with a null hypothesis of a uniform distribution across all best IPDs. The data were consistently significantly non-uniformly distributed below the 1000–1200 Hz band (p-values: 0–300 Hz p = 2.7×10^−13^, 300–400 Hz p = 1.1×10^−5^, 400–500 Hz p = 1.6×10^−5^, 500–600 Hz p = 0.024, 600–700 Hz p = 5.5×10^−4^, 700–800 Hz p = 0.038, 800–1000 Hz p = 0.013. n = 128 in all cases), but not significantly different from uniform at and above this band (1000–1200 Hz, 1200–1400 Hz, 1400–2300 Hz, all N.S., n = 128 in all cases). This is largely consistent with the predictions of the optimal-coding model, where the transition between distinct sub-populations and uniformity is suggested to occur around 1000 Hz (Q3 confirmed). It is worth considering the possibility that there may still be some slight non-uniformity at the high frequencies that is below the power of the test. Indeed, a variation on the optimal-coding model [26] predicts slight non-uniformities at high frequencies.

In summary, across the five species: For the intermediate- to large-sized species (cat, guinea pig, and macaque), the neural representation of the major auditory spatial cue (the distribution of best IPDs) shows distinctly different coding regimes at different sound frequencies within the same species. Furthermore, the neural representation also depends on the head size, with smaller mammals having a two-channel representation at all frequencies, unlike intermediate-sized mammals. Throughout, the distributions of best IPDs depend on head size and sound frequency in a systematic manner, as predicted by the optimal-coding model.

## Discussion

Overall, the data indicate that many mammalian species employ multiple different neural representations of the auditory spatial cue of interaural time difference, with the representation used depending on head size and sound frequency. This provides cross-species, experimentally derived, support for the optimal-coding model of ITD, and suggests that the classic ‘Jeffress’ model and the more-recently promoted two-channel model represent limiting cases of the optimal-coding model, at high sound frequencies and large head sizes, and at low sound frequencies and small head sizes respectively. At low normalized frequencies (sound frequency as a proportion of 1/maximum ITD for each species), the majority of best IPDs recorded experimentally lie beyond the physiological range. This pattern was present, and statistically significant, across all the mammalian species examined, from small desert-dwelling rodents to large primates (addressing Q1). At intermediate normalized frequencies, in the relevant species (guinea pig, cat, and macaque), a central subpopulation is observed in the lower intermediate range of frequencies, but a significant dip in the distribution (indicating bimodality) is observed in the higher intermediate range (addressing Q2). At high normalized-frequencies (in the macaque, where sufficient data were obtained), best IPDs are more uniformly distributed (addressing Q3). The predictions of Q2 and Q3 were entirely novel, generated by the optimal-coding model purely from theoretical considerations, and subsequently confirmed (in all cases with sufficient data) in analysis of previously-obtained experimental data. Of particular note are the central subpopulations in the intermediate range, and the existence of multiple different forms of distribution across the ITD-sensitive frequency range of the guinea pig, cat, and macaque. Additionally, the prediction of bimodal distributions of IPD detectors at the lowest sound-frequencies had not been reported for the macaque. In general, the guinea pig, cat, and macaque show multiple, different distributions, arranged in a specific order as a function of ascending frequency bands, whereas the gerbil and kangaroo rat show only bimodality in their distributions.

Despite general agreement with the optimal-coding model, there are some, mostly quantitative, aspects of the observed distributions of best IPDs that are not predicted. First, best IPDs are more scattered in the data than in the model, with the subpopulations appearing more as peaks in a distribution than discrete points. This scatter may be due to the inherent variability of biological systems, or perhaps to an increase in the degree of heterogeneity in the neural responses to better deal with variation in other stimulus dimensions, or in order to carry out coding tasks not requiring discrimination [41]. Nevertheless, the overall frequency-dependent positioning of the peaks in the distribution of best IPDs is consistent with the optimal-coding model (across a wide range of species), indicating the importance of precise IPD estimation and discrimination for these neurons. Second, in some cases, certain minor subpopulations that were predicted by the model were not observed in the data, such as the central sub-populations at 800–1000 Hz in the macaque. Third, the optimal-coding model does not predict with total accuracy the IPDs on which some subpopulations were centred. This may be due, among other factors, to the shape of rate-vs.-IPD curves in the optimal-coding model not exactly matching those actually present in the animal. Finally, the frequencies at which transitions between subpopulations of best IPDs become apparent differ slightly between the data and the model. Macaques and guinea pigs show transitions at slightly lower frequencies than predicted. This may indicate that a larger maximum ITD occurs in natural environments (such as in near-field or multi-source environments) than the values provided by current measurements. Alternatively, it may indicate IPD tuning curves in the macaque cortex, at the relevant sound frequencies, are sharper than those used in the model. In contrast, cats show these transitions at higher frequencies in the data than in the model. A possible reason as to why cats, with similar maximum ITDs to guinea pigs, show transitions in subpopulation regimes at somewhat higher frequencies, is that they, being predators, may concentrate their auditory spatial capacities towards the midline. This effect is often seen in the senses of predators, e.g. forward-facing eyes, tight visual fovea, etc. That is, they devote more coding resources to the representation of a narrow range of ITDs around the midline, at the expense of representation of more peripheral ITDs. This ‘auditory spatial fovea’ would mean they effectively have a smaller maximum IPD.

In conclusion, we examined experimentally recorded distributions of neural tuning for ITD across five mammalian species. We find that for high frequencies and large-headed mammals, distributions exhibit characteristics of the Jeffress model. Additionally, for low frequencies and small-headed mammals the distributions exhibit characteristics consistent with the two-channel model. However, in general, these two models are inconsistent with the full range of representations of ITD observed across different sound frequencies and head sizes. In contrast, the optimal-coding model, of which the above two models can be seen as particular instantiations at certain sound frequencies, is a general model motivated by a principled theoretical framework by which differences in the distributions of neural tuning functions across species might be understood. Crucially, the optimal-coding model explains the new experimental findings we present; for intermediate head-sizes we find different types of neural representation of ITD for different sound frequencies in the same species, including novel representations not previously observed. This suggests that the optimality approach, whether employing the model used here [26] or through yet-to-be-determined optimality models, has the capacity to explain many aspects of the form of the neural code by which ITD is represented across species. These findings further the case for normative approaches providing general cross-species principles underpinning neural systems. Furthermore, they raise the general point that, for sensory systems, the form of neural representation may change extensively and sharply for different species, and even for different stimuli of the same modality within the same animal.

## Materials and Methods

### Experiments and preliminary analyses

The guinea pig data was gathered in the UK, and the macaque data in the US, in different labs as part of entirely separate studies that were later synthesized into this paper. Thus the macaque and guinea pig data were governed by separate ethical protocols.

#### Guinea pig

Single-neuron recordings were made from the inferior colliculus of urethane-anesthetized guinea pigs (*Cavia porcellus*) using glass-coated tungsten microelectrodes. These data contributed to a range of different studies investigating binaural hearing [21], [32], [33]. All experiments were carried out in accordance with the Animal (Scientific Procedures) Act of 1986 of Great Britain and Northern Ireland. All procedures were reviewed and approved under UK Home Office Licence (covered by both Project and Personal licenses). At the end of each experiment, the guinea pig was sacrificed with an overdose of sodium pentobarbital by intraperitoneal injection.

After isolation of a neuron, each neuron’s characteristic frequency (CF) was determined. The CF is the sound frequency with the lowest sound-intensity threshold, and is typically close to the best frequency (BF), the sound-frequency that elicits the greatest firing rate for a given sound intensity. The CF was determined audio-visually using binaurally-presented tones with zero ITD, and then by a detailed frequency-versus-level response area covering a 6-octave range around this CF. Noise stimuli to measure noise-delay (firing rate vs. ITD) functions consisted of identical (frozen) broadband noise bursts (50 Hz–5 kHz) presented dichotically to each ear using 12.7-mm Brüel and Kjær (Nærum) condenser ear- phones at 10–20 dB above the neuron’s noise threshold. Noise-delay functions were constructed over a range of ITDs equal to 3 times the period of the neuron’s CF. Either 20 repetitions of a 50-ms burst of noise, or 3 repetitions of a 320-ms burst of noise were presented at each of 51 equally-spaced delays over this range. For each neuron, a sinusoid was fitted to the neuron’s rate-vs-ITD function to obtain a measure of best ITD. Since noise rather than tones was employed as a stimulus, best IPD was considered to be the best ITD divided by the period of the best-fitted sinusoid, which approximates best IPD to a pure tone [22]. The frequency of this sinusoid corresponds to the dominant frequency component in the response, and will closely match the BF [33], and provides the frequency measures in Figure 2H.

#### Macaque

Subjects were two male rhesus monkeys (*Macaca mulatta*). The responses of 248 single neurons were recorded from the low-frequency auditory core (AI and R) of both hemispheres during awake passive listening. The physiological techniques have been described previously [40], [42], and all procedures involving animal use and welfare in this study were reviewed and approved by the New York University Institutional Animal Care and Use Committee. Animals were pair-housed at the NYU Animal Facility, kept on a 12-hour light/dark cycle, and allowed access to a variety of enrichment activity (mirrors, toys, etc.). Food (monkey biscuits) was available ad-lib, and fresh fruit and other treats were given daily following each session. During experimental sessions, animals performed an auditory task of discriminating IPDs to earn liquid reward, and were allowed to work to satiety. The neural recordings used were made when the macaques were in a passive but awake state between behavioural sessions. To maintain alertness, animals were monitored by video and given periodic rewards between stimulus sets. For the implant surgery (under sterile conditions) that allowed for the neural recording, anaesthesia was induced using ketamine and sodium thiopental. A surgical plane maintained with isoflurane. These data contributed to a range of different studies investigating binaural hearing. At the conclusion of a series of studies from which these data were drawn, animals were deeply anesthetized by intravenous injection of sodium pentobarbital, then transcardially perfused. Post-mortem, standard histological processing verified the recording sites to be within core auditory cortex.

For the data presented in the results the responses to IPDs were measured using binaural beats, a continuous modulation of IPD produced when the tone frequency presented to one ear differs slightly from the frequency presented to the other. The stimulus traverses 360° of interaural phase at a rate determined by the frequency mismatch (here, a period of 500 ms for a 2-Hz difference in frequency). Beats were presented across multiple carrier frequencies spanning the receptive field of each neuron in typically 100-Hz steps, at each neuron’s best sound pressure level (median 60 dB SPL). Neural discharges were folded on the beat cycle, from which synchrony to the beat (i.e. tuning to IPD) was measured by vector strength. The best IPD was determined by the mean phase of the spike discharges, if synchrony to the beat was significant by the Rayleigh test (p<1×10^−3^).

#### Other species

For cat, kangaroo rat, and gerbil, data were extracted from published figures in the relevant papers [18], [19], [25], [28], [37] using the programs Techdig or PlotDigitizer. In the kangaroo rat (*Dipodomys spectabilis*), Crow et al. [18] examined the distribution of 51 neural best ITDs to interaurally-delayed tones as a function of frequency, for 28 ITD-sensitive neurons (some neurons were measured at multiple frequencies), recorded in the superior olivary complex, the presumed site of primary binaural integration. In the gerbil (*Meriones unguiculatus*), Pecka et al. [28] measured best ITD as a function of best frequency (the frequency to which a neuron responds most) in the medial superior olive, the dominant ITD-sensitive nucleus of the superior olivary complex, using pure tones or narrowband noise. In the cat (*Felis catus*), Hancock and Delgutte [19] and Joris et al. [37] measure best ITDs to interaurally-delayed noise as a function of neural best frequency and the similar measure of characteristic frequency respectively. For all species we converted the best ITDs to best IPDs by dividing by the period of the sound frequency.

### Modelling

The optimal-coding model postulates that best ITDs are arranged to represent most precisely ITDs within the physiological range, conditioned on head size, and given limitations such as neural noise. This pan-species model is motivated by principles of efficient coding [43], [44], which hypothesises that, through natural selection, neural representations are optimized to be efficient for coding of natural stimuli. The current study aims to test experimentally a key prediction of the optimal-coding model; namely, that many species exhibit different representations of ITD at different sound frequencies. For a full description of the optimal-coding model see Harper & McAlpine [26].

Briefly, the optimal-coding model was developed to predict the optimal distribution of a population of neural best ITDs that represents most precisely the ITD of a pure tone, for all ITDs within the physiological range (i.e. for ITDs up to the maximum ITD). For a given species and pure-tone frequency, the optimal distribution of best ITDs is found by varying the best ITDs of a population of model neurons so as to minimise a measure of coding error for ITD. The firing rate-vs.-ITD function of a model neuron, and its best ITD, are illustrated in Figure 1C. In this, and all subsequent figures, ITD is considered in terms of the equivalent interaural phase difference (IPD), i.e. the ITD as a proportion of the period of pure tone frequency. The optimal-coding model predicts the distribution of best IPDs (best ITD in terms of equivalent IPD) for all the neurons that respond to a given sound frequency. Practically, however, this response will be dominated by those neurons for which the sound frequency represents their best frequency. Therefore, it is the distribution of best IPD over best frequency will also be appropriate to compare with the optimal-coding model’s predictions. For gerbil, guinea pig, and cat, BF (or a similar measure) was employed, and for macaque and kangaroo rat stimulating frequency (although with typically only a few frequencies per neuron). This decision was based entirely on the format in which the data were available. Furthermore, the best ITD to broadband noise tends to be similar to that to a pure tone at BF [22], so either is used depending on data availability. Finally, the model employs sharpened cosine functions to represent rate-vs.-IPD curves, which although describing well most ITD functions, do not account for the shape of a relatively small proportion (10–20%) of ITD-sensitive neurons referred to as ‘trough-type’ neurons [45], [46]. Thus, where possible, only the distribution of best IPDs for ‘peak-type’ neurons – neurons that accord with the form posited in the Jeffress model - was assessed with respect to the optimal-coding model.

Figure 1D illustrates the general predictions of the optimal-coding model. Predictions of the optimal-coding models are: at the lowest frequencies, a “two-channel” representation exists with best IPDs largely lying outside the physiological range. At low intermediate frequencies, a central sub-population of best IPDs with flanking sub-populations exists. At high intermediate frequencies, again a “two-channel” representation exists but with best IPDs inside the physiological range. Above this two-channel representation, a central population re-emerges over a narrow, higher, frequency range. However, for most species, an insufficient number of IPD-sensitive neurons with BFs in the range were recorded to examine this perspective. At the highest frequencies a uniform/unimodal distribution of best IPDs exists, consistent with the Jeffress model. Of course, any sub-populations in the data would be expected to be substantially more diffuse than in the model, with the sub-populations in the data being peaks in the distribution of best IPDs.

An essential parameter of the optimal-coding model is the maximum IPD – the maximum ITD divided by the period of that pure-tone frequency. The maximum IPD refers to the limit of the physiological range of ITDs, expressed in terms of IPD, at any given frequency. The maximum IPD can also be considered as the frequency normalized by 1/maximum ITD, this is the normalized frequency in Figures 1D–F. We employ the maximum ITD, as determined experimentally, whenever possible (for all species except the kangaroo rat), rather than estimates from the interaural distance. The distribution of best IPDs predicted for a species with a given maximum ITD and for a given sound frequency can be obtained from Figure 1D by examining the distribution at the corresponding normalized sound frequency on the ordinate. For each species, the maximum ITD is set (although see below), and the ordinate is then denoted as un-normalized sound frequency (see Figures 2A, D, G, I, 3A–B). The predictions for each species, therefore, represent scaled versions of the predictions of the general model in Figure 1D. To facilitate comparison with the data, the model predictions for specific species (Figures 2A, D, G, I, 3A–B) are averaged together over the same large frequency and IPD bins as employed in the data. Also, to further facilitate comparison, the distribution of ITDs in each large frequency band is scaled such that, in each band, the maximum number of best IPDs in an IPD bin matches that of the data, as the model makes no assumptions as to the proportion of neurons in each frequency band.

One minor complication is that maximum ITD can vary slightly with sound frequency (up to about 25%). Accounting for this in the optimal-coding model results in a slight non-linear rescaling over frequency of the general model in Figure 1D. For those animals for which maximum ITD as a function of frequency is available - the guinea pig [29], the cat [30], and the macaque [38] - we used the frequency-dependent maximum ITD. The maximum ITD as a function of frequency was extracted from the relevant papers using PlotDigitizer, linearly interpolated, and where necessary extrapolated by continuing at the endpoint maximum ITD. The frequency dependence of the maximum ITD has little effect on the model predictions, as the maximum ITD fluctuations are small.

An important value in the current study is the ‘specific frequency’, which refers to the lowest frequency at which a sub-population of best IPDs within the physiological range appears in the optimal-coding model. This occurs where the maximum IPD (the normalized frequency) is approximately equal to 0.12. For a given species, the specific frequency is equal to 0.12 divided by the maximum ITD (measured in seconds). For the gerbil this is 1000 Hz (0.12/0.000120 s), for the kangaroo rat 1143 Hz (0.12/0.000105 s). For the animals where frequency dependent maximum ITD is used, the specific frequency is the lowest frequency where the maximum IPD is 0.12; for the guinea pig 369 Hz, cat 387 Hz, and for the macaque 209 Hz.

### Analysis

For each species, the experimentally recorded data are represented in the form of a mirrored 2D histogram showing the number of neurons with particular best IPDs as a function of frequency. Mirroring the data assumes that the brain is symmetric across the midline and that, consequently, each neuron has a partner neuron in the equivalent contralateral brain region whose best IPD is of equal magnitude and opposite sign. This assumption permits an estimation of the distribution of best IPDs even when they were not equally sampled from both sides of the brain. Mirroring was only used for display, not for statistical analysis. Bin sizes for each frequency were chosen to be round numbers (300, 250, and 200 Hz) of appropriate size in order ensure a reasonable number of data points in each bin, whilst still making it possible to observe any cross- frequency patterns in the data. Bin sizes were kept constant for similarly-sized animals with similarly-sized data sets. IPD bin-sizes were always 0.05 cycles. Best ITDs were converted to IPD by dividing by the stimulation frequency or the neuron’s best frequency (or a similar measure). In the small number of cases where ITDs were greater in magnitude than half a cycle, ITDs were wrapped back by a whole number of cycle of IPD to ensure they fell between −0.5 to 0.5 cycles.

Sufficient data permitting (statistical tests only when n>10), for data for each of the five species, the form of the distributions of best IPDs over frequency was analyzed. The results were then compared to the predictions of the optimal-coding model, and also the Jeffress model and two-channel model. Specifically, the following questions were addressed:

Q1) At low frequencies (normalized frequency below ∼0.12), does the distribution of best IPDs fall largely outside the physiological range (i.e. consistent with a ‘two-channel’ representation), as predicted by the optimal-coding model?

Although this prediction has been reported some small mammals, it has not been tested in a systematic manner across species and remains contentious. We address this for each species by applying a binomial test to the experimentally recorded data in this frequency range. The null hypothesis is that a greater or equal number of best ITDs lie within the physiological range than beyond. If the null hypothesis is rejected, then a (statistically significantly) greater number of best IPDs lie beyond the physiological range than within. Note that the null hypothesis represents a conservative measure of accordance with the Jeffress model, allowing up to half of neurons to show best ITDs beyond the physiological range of ITDs generated by the head.

Q2) At intermediate frequencies (normalized frequency ∼0.12 to ∼0.5), do the data indicate the novel distributions of best IPDs predicted by the optimal-coding model - a central peak in the distribution in the lower intermediate frequencies and, above that, a bimodal distribution within the physiological range?

Neither the Jeffress model nor the two-channel model would predict such an outcome, and this prediction is untested in any species to date. Examining the bimodal distribution of the optimal-coding model within the physiological range, we observe that the sub-populations fall beyond 0.075 cycles IPD (Figure 1. This is also true of the two-channel model at all frequencies Thus, we use a binomial test for the data in each frequency band covering the above intermediate frequency range. For each band, the null hypothesis (consistent with a Jeffress-like distribution) is that the number of neurons with best IPDs over the range 0–0.075 cycles IPD in magnitude is greater than or equal to the number over the range 0.075–0.15 cycles IPD in magnitude. That is, those frequencies are determined where a significant narrow central dip exists in the distribution of IPDs.

Q3) At high frequencies (normalized frequency above ∼0.5), do the data tend toward a uniform or unimodal best-IPD distribution (i.e. is the distribution Jeffress-like), as predicted by the optimal-coding model?

This prediction has not yet been examined in mammals. The macaque is the only species from which sufficient data are available over a sufficiently-high range of sound frequencies range to conduct this analysis. For the macaque, the Kuiper’s Test is applied to each frequency band, with a null hypothesis of a uniform distribution across over the IPD cycle. By this method, the frequency at which the distribution of best IPDs becomes indistinguishable from uniform can be determined, which is then compared with the optimal-coding model.

## References

[1] Rayleigh L (1907) On our perception of sound direction. Philos Mag 13: 214–232.

[2] Wightman FL, Kistler DJ (1992) The dominant role of low-frequency interaural time differences in sound localization. J Acoust Soc Am 91: 1648–1661. 1564201 10.1121/1.402445

[3] Jeffress LA (1948) A place theory of sound localisation. J Comp Physiol Psychol 41: 35–39. 18904764 10.1037/h0061495

[4] Colburn HS (1977) Theory of binaural interaction based on auditory-nerve data. II. Detection of tones in noise. J Acoust Soc Am 61: 525–533. 845314 10.1121/1.381294

[5] Stern RM Jr, Colburn HS (1978) Theory of binaural interaction based in auditory-nerve data. IV. A model for subjective lateral position. J Acoust Soc Am 64: 127–140. 711991 10.1121/1.381978

[6] Bilsen FA (1977) Pitch of noise signals: evidence for a “central spectrum”. J Acoust Soc Am 61: 150–161. 833366 10.1121/1.381276

[7] Frijns JH, Raatgever J, Bilsen FA (1986) A central spectrum theory of binaural processing. The binaural edge pitch revisited. J Acoust Soc Am 80: 442–451. 3745676 10.1121/1.394040

[8] Raatgever J, Bilsen FA (1977) Lateralization and dichotic pitch as a result of spectral pattern recognition. In: Evan EF, Wilson JP, editors. Psychophysics and Physiology of Hearing. London: Academic Press. pp. 443–453.

[9] Raatgever J, Bilsen FA (1986) A central spectrum theory of binaural processing. Evidence from dichotic pitch. J Acoust Soc Am 80: 429–441. 3745675 10.1121/1.394039

[10] Stern RM, Zeiberg AS, Trahiotis C (1988) Lateralization of complex binaural stimuli: a weighted-image model. J Acoust Soc Am 84: 156–165. 3411043 10.1121/1.396982

[11] Sayers BM, Cherry C (1957) Mechanisms of binaural fusion in the hearing of speech. J Acoust Soc Am 29: 973–987.

[12] Colburn HS (1996) Auditory Computation. In: Hawkins HL, McMullen TA, Popper AN, Fay RR, editors. Auditory Computation. New York, NY: Springer-Verlag. pp. 332–400.

[13] Coles RB, Guppy A (1988) Directional hearing in the barn owl (Tyto alba). J Comp Physiol A 163: 117–133. 3385664 10.1007/BF00612002

[14] Takahashi T, Konishi M (1986) Selectivity for interaural time difference in the owl's midbrain. J Neurosci 6: 3413–3422. 3794779 10.1523/JNEUROSCI.06-12-03413.1986 PMC6568656

[15] Wagner H, Takahashi T, Konishi M (1987) Representation of interaural time difference in the central nucleus of the barn owl's inferior colliculus. J Neurosci 7: 3105–3116. 3668618 10.1523/JNEUROSCI.07-10-03105.1987 PMC6569176

[16] Joris PX, Smith PH, Yin TC (1998) Coincidence detection in the auditory system: 50 years after Jeffress. Neuron 21: 1235–1238. 9883717 10.1016/s0896-6273(00)80643-1

[17] Brand A, Behrend O, Marquardt T, McAlpine D, Grothe B (2002) Precise inhibition is essential for microsecond interaural time difference coding. Nature 417: 543–547. 12037566 10.1038/417543a

[18] Crow G, Rupert AL, Moushegian G (1978) Phase locking in monaural and binaural medullary neurons: implications for binaural phenomena. J Acoust Soc Am 64: 493–501. 712011 10.1121/1.381999

[19] Hancock KE, Delgutte B (2004) A physiologically based model of interaural time difference discrimination. J Neurosci 24: 7110–7117. 15306644 10.1523/JNEUROSCI.0762-04.2004 PMC2041891

[20] Jiang D, McAlpine D, Palmer AR (1997) Responses of neurons in the inferior colliculus to binaural masking level difference stimuli measured by rate-versus-level functions. J Neurophysiol 77: 3085–3106. 9212259 10.1152/jn.1997.77.6.3085

[21] McAlpine D, Jiang D, Palmer AR (2001) A neural code for low-frequency sound localization in mammals. Nat Neurosci 4: 396–401. 11276230 10.1038/86049

[22] Palmer AR, Rees A, Caird D (1990) Interaural delay sensitivity to tones and broad band signals in the guinea-pig inferior colliculus. Hear Res 50: 71–86. 2076985 10.1016/0378-5955(90)90034-m

[23] Palmer AR, Rees A, Caird D (1992) Binaural masking and sensitivity to interaural delay in the inferior colliculus. Philos Trans R Soc Lond B Biol Sci 336: 415–422. 1354383 10.1098/rstb.1992.0077

[24] Siveke I, Pecka M, Seidl AH, Baudoux S, Grothe B (2006) Binaural response properties of low-frequency neurons in the gerbil dorsal nucleus of the lateral lemniscus. J Neurophysiol 96: 1425–1440. 16571733 10.1152/jn.00713.2005

[25] Stillman RD (1971) Characteristic delay neurons in the inferior colliculus of the kangaroo rat. Exp Neurol 32: 404–412. 5110223 10.1016/0014-4886(71)90007-0

[26] Harper NS, McAlpine D (2004) Optimal neural population coding of an auditory spatial cue. Nature 430: 682–686. 15295602 10.1038/nature02768

[27] Maki K, Furukawa S (2005) Acoustical cues for sound localization by the Mongolian gerbil, Meriones unguiculatus. J Acoust Soc Am 118: 872–886. 16158644 10.1121/1.1944647

[28] Pecka M, Brand A, Behrend O, Grothe B (2008) Interaural time difference processing in the mammalian medial superior olive: the role of glycinergic inhibition. J Neurosci 28: 6914–6925. 18596166 10.1523/JNEUROSCI.1660-08.2008 PMC6670983

[29] Sterbing SJ, Hartung K, Hoffmann KP (2003) Spatial tuning to virtual sounds in the inferior colliculus of the guinea pig. J Neurophysiol 90: 2648–2659. 12840079 10.1152/jn.00348.2003

[30] Roth GL, Kochhar RK, Hind JE (1980) Interaural time differences: implications regarding the neurophysiology of sound localization. J Acoust Soc Am 68: 1643–1651. 7462463 10.1121/1.385196

[31] Kuwada S, Fitzpatrick DC, Batra R, Ostapoff EM (2006) Sensitivity to interaural time differences in the dorsal nucleus of the lateral lemniscus of the unanesthetized rabbit: comparison with other structures. J Neurophysiol 95: 1309–1322. 16338997 10.1152/jn.00901.2005

[32] McAlpine D, Jiang D, Palmer AR (1996) Interaural delay sensitivity and the classification of low best-frequency binaural responses in the inferior colliculus of the guinea pig. Hear Res 97: 136–152. 8844194

[33] Marquardt T, McAlpine D (2006) A ð-limit for coding ITDs: Implications for binaural models. In: Kollmeier B, Klump G, Hohmann V, Langemann U, Mauermann M et al.., editors. Hearing - From Sensory Processing to Perception. Heidelberg: Springer. pp. 407–416.

[34] Kuwada S, Stanford TR, Batra R (1987) Interaural phase-sensitive units in the inferior colliculus of the unanesthetized rabbit: effects of changing frequency. J Neurophysiol 57: 1338–1360. 3585471 10.1152/jn.1987.57.5.1338

[35] Yin TC, Kuwada S, Sujaku Y (1984) Interaural time sensitivity of high-frequency neurons in the inferior colliculus. J Acoust Soc Am 76: 1401–1410. 6512102 10.1121/1.391457

[36] Yin TC, Kuwada S (1983) Binaural interaction in low-frequency neurons in inferior colliculus of the cat. II. Effects of changing rate and direction of interaural phase. J Neurophysiol 50: 1000–1019. 6631458 10.1152/jn.1983.50.4.1000

[37] Joris PX, Van de Sande B, Louage DH, van der Heijden M (2006) Binaural and cochlear disparities. Proc Natl Acad Sci U S A 103: 12917–12922. 16908859 10.1073/pnas.0601396103 PMC1568946

[38] Spezio ML, Keller CH, Marrocco RT, Takahashi TT (2000) Head-related transfer functions of the Rhesus monkey. Hear Res 144: 73–88. 10831867 10.1016/s0378-5955(00)00050-2

[39] Moore BCJ (2003) An Introduction to the Psychology of Hearing, 5th Edition. San Diego, CA: Academic Press.

[40] Scott BH, Malone BJ, Semple MN (2007) Effect of behavioral context on representation of a spatial cue in core auditory cortex of awake macaques. J Neurosci 27: 6489–6499. 17567810 10.1523/JNEUROSCI.0016-07.2007 PMC6672434

[41] Brette R (2010) On the interpretation of sensitivity analyses of neural responses. J Acoust Soc Am 128: 2965–2972. 21110592 10.1121/1.3488311

[42] Scott BH, Malone BJ, Semple MN (2009) Representation of dynamic interaural phase difference in auditory cortex of awake rhesus macaques. J Neurophysiol 101: 1781–1799. 19164111 10.1152/jn.00678.2007 PMC2695633

[43] Attneave F (1954) Some informational aspects of visual perception. Psychol Rev 61: 183–193. 13167245 10.1037/h0054663

[44] Barlow H (1961) In: Rosenblith W, editor. Sensory Communication. Cambridge, MA: MIT Press. pp. 217–234.

[45] Agapiou JP (2007) Asymmetry in neural responses to interaurally time delayed stimuli. Ph.D. Thesis. University College London.

[46] McAlpine D, Jiang D, Shackleton TM, Palmer AR (1998) Convergent input from brainstem coincidence detectors onto delay-sensitive neurons in the inferior colliculus. J Neurosci 18: 6026–6039. 9671687 10.1523/JNEUROSCI.18-15-06026.1998 PMC6793065

